# Language as a marker of ethnic identity among the Yucatec Maya

**DOI:** 10.1017/ehs.2020.39

**Published:** 2020-06-29

**Authors:** Cecilia Padilla-Iglesias, Robert A. Foley, Laura A. Shneidman

**Affiliations:** 1 Leverhulme Centre for Human Evolutionary Studies, Department of Archaeology, University of Cambridge, Cambridge, UK; 2 Department of Anthropology, University of Zurich, Zurich, Switzerland; 3 Department of Psychology, Pacific Lutheran University, Washington, USA

**Keywords:** Essentialism, developmental plasticity, cultural markers, language, Mexico, evolution

## Abstract

Most human variation is structured around symbolically marked cultural (‘ethnic’) groups that require common codes of communication. Consequently, many have hypothesised that using others’ linguistic competences as markers of their descent is part of an evolved human psychology. However, there is also evidence that the use of language as ethnic markers is not universally applied, but context specific. We explore the tension between these views by studying responses to bilingualism among 121 adults living in Mayan communities undergoing rapid socioeconomic changes involving increased contact with Spanish-speaking towns. We show that, although competences in Mayan were strongly tied to perceiving others as having a Mayan ethnic identity, ethnolinguistic category membership was not seen as stable through life, vertically transmitted, nor regarded as incompatible with competences in Spanish. Moreover, we find variation in how people reasoned about ethnolinguistic identities depending on their *own* linguistic repertoires. Our work suggests that, while there may be an evolved predisposition to use language as a signal of group identity, our developmental plasticity allows us to respond adaptively to social information around us, leading to psychological and behavioural variation within and across populations. How people reason about others based on their linguistic profiles will affect the payoffs of acquiring different languages and ultimately the long-term sustainability of linguistic diversity.

## Graphical abstract

**Figure:**
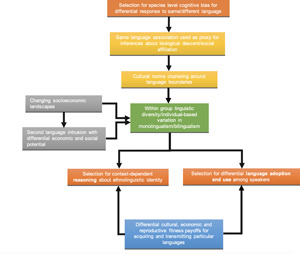

**Media summary:** Evolutionary approach to language use shows how Mayan identity is both maintained and altered by bilingualism following market integration.

## Introduction

Humans are the only species whose social organisation is structured around symbolically marked cultural (ethnic) groups, where members tend to share norms, expectations, skills and beliefs (e.g. Richerson & Boyd, 2001; Foley and Lahr, 2011). Given that ethnic groups require common codes of communication, researchers have proposed that linguistic variation has played a primary role throughout our evolutionary trajectory in creating, preserving and indexing such variation between human populations (Atkinson et al., 2008; Moya & Henrich, 2016; Cohen, 2012; Dunbar, 2003).

On the one hand, by limiting between-group communication, linguistic boundaries restrict the diffusion of institutions between societies, further enhancing cultural differences between populations (Richerson et al., 2016; Perreault, 2012; Bell et al., 2009). On the other, the landscapes emerging from such clustering of cultural institutions around linguistic boundaries would have lain at the foundation of our social psychology, in particular in the way humans use linguistic variation to reason about others’ social group membership or guide their social behaviours towards them (Nettle & Dunbar, 1997; Dunbar, 2003; Cohen, 2012; Moya & Henrich, 2016; Shutts et al., 2009). Selection pressures acting on our linguistic codes also lie at the heart of many theories attempting to explain the evolution of our species’ complex communicative codes, or their diversity (e.g. Allen et al., 2011; Nettle, 1998; Currie & Mace, 2009).

Assuming that language boundaries match those of cultural (ethnic) groups, one hypothesis is that humans have evolved to use linguistic features as predictive of ethnic category membership (Moya, 2013). Kinzler and Spelke (2007) argue that language is such an important marker of in-group status (and much more historically reliable than physical features) that it could be a candidate for a core (and therefore innate) knowledge domain. In turn, displaying homophilic preferences based on language could have been selectively advantageous, avoiding coordination costs when interacting with other group members (McElreath et al., 2003; Jensen et al., 2015; Chudek & Henrich, 2011). There is extensive ethnographic evidence that ethno-linguistic boundaries often delineate institutions such as reciprocity, marriage, defence alliances or risk-sharing networks (Wiessner, 1983; Nettle, 1998; Currie & Mace, 2009; Thomason, 2007), and that ideologies about language use motivate social distancing, stereotyping and political action (Schieffelin et al., 1998; Irvine et al., 2000). Moreover, in laboratory experiments, US infants as early as 5 months show a looking preference, and by 10 months prefer to befriend or exchange toys with native-language speakers (Kinzler et al., 2009, 2011, 2012). These observations would suggest that using language as a group marker is an evolved cognitive trait.

However, many societies worldwide are undergoing rapid socioeconomic changes that involve exposure to foreign languages, and so the greater occurrence of bi- and multilingualism. While these opportunities have probably always occurred, the scale of socioeconomic change is now much greater (Kandler et al., 2010; Isern & Fort, 2014). This changes the predictive value of language as a cultural marker, and opens up tactical and strategic opportunities and challenges for individuals and groups. Language and language use can remain a marker, but one that requires a more flexible response. In such contexts, what happens when speakers become bi- or multilingual? If language is an important marker of ethnic category membership, learning an additional language should introduce noise and/or be regarded as an attempt to abandon one's ethnic affiliation (and consequently culture-specific institutions, norms and behaviours) in exchange for another (Lambert, 1981). If that is the case, acquiring bilingual competences (even if one still keeps hold of the local language) could even be culturally sanctioned. That is, there could be social costs associated with becoming bilingual, which could translate into negative fitness consequences (Henrich, 2017; Padilla-Iglesias and Kramer, in press).

This is consistent with a broader evolutionary and ecological perspective. Behaviours and traits exist in a cost–benefit matrix; the acquisition of a second language is usually seen as entirely beneficial, but it comes with costs as well. These go beyond the cognitive, time and opportunity costs of learning the language and include a more negative perception by others. Among many societies individuals who attempt to gain status by a change in behaviour may be ostracised or socially excluded (Lee, 1988).

A famous example of these ‘social costs to bilingualism’ is described by Aikhenvald (2003) with regards to the Vaupés basin in Brazil. In this area, linguistic affiliation serves as an indicator of descent and guides marriage patterns, with explicit rules on who has or has not the right to speak particular languages. Individuals may (and are expected to) become bilingual in their ‘native’ local languages and the local lingua franca, Tucano. However, if they use a language that delineates another descent group, this is heavily frowned upon. Moreover, in this region, using Portuguese (the national language) is associated with the negative image of an Indian who tries to be better than his peers, and has very negative effects on access to local social networks (Aikhenvald, 2006).

Children tend to learn language from those individuals who are around them during the early phases of development (the so-called ‘critical period’ for language acquisition; see Hurford, 1991; Best et al., 2001). Therefore, another possibility is that linguistic affiliation simply acts as a probabilistic badge of relatedness and thus people have evolved to use it to guide cooperation based on self-similarity (kin-based altruism) instead of on a shared cultural repertoire (Dunbar et al., 1999; Dunbar, 2003). This would require ‘ethnolinguistic identities’ to not only be mutually exclusive from one another, but also relatively fixed through life, and always acquired ontogenetically early in development (Allen et al., 2011). Indeed, young European-American children expect language identity to be inherited from birth parents rather than from their social context and infer that language use is more stable through the life course than race is (Kinzler & Dautel, 2012). Hence, at least experimentally, American pre-schoolers treat language use as predictive of skin colour, residence and clothing (Hirschfeld & Gelman, 1997).

These multiple studies show that beyond the hypothesis that the use of language is a group marker, accommodation must be made for other factors that could add greater dynamism (Tajfel, 1979; Giles, 1977). They also highlight further questions that need to be addressed concerning *which* language speakers should use to convey their group member and how exactly should listeners reason about the meaning of the signal. In other words, whilst there is evidence for a long-term cognitive bias towards shared first language as a basis for prediction of social group membership, there is also support for a more pragmatic and context-dependent use in specific situations such as where bilingualism occurs, and that the link between the two is dependent upon a process of reasoning and inference about the social information signalled by language use (Jensen et al., 2015; Moya & Boyd, 2015; Cohen & Haun, 2013; Hill, 1978). Two important elements of those situations are rapid socioecological change and bilingualism. Both of these may be expected to influence how individuals reason about group identity based on language competence.

This paper addresses these broad issues, and in particular how bilingualism, driven by different economic and cultural spheres of influence, affects reasoning about the relationship between language and group identity, and thus impacts broader inferences about language–ethnicity covariation. Since evolution can more broadly select for diversity in developmental patterns as a means for providing adaptive plasticity, the aim here is to see whether inter-individual differences in reasoning about ethnolinguistic identity are the strategic equivalent of this process. In doing so, we hope to shed light on why there may be evolutionary payoffs for flexibility in the domain of reasoning about linguistic identity in the context of a changing ecology. The research context is Yucatec Mayan communities, which are undergoing rapid socioeconomic changes as a result of increased access to education, wage labour and connectedness with urban centres, where the language spoken is Spanish. More and more people are acquiring bilingual competences in Spanish as well as the local indigenous language (Yucatec Mayan). The fieldwork investigated whether linguistic competences are regarded by the Mayan communities as a source of useful social information, concretely about cultural or ethnic identity and/or relatedness. Key research questions are whether different language competences, such as degree of bilingualism, influence perceptions of identity; how these are inherited; how stable they are through life; and how these vary with individual attributes such as sex, age or status. The focus is on whether, and if so, how, people can flexibly adapt their way of reasoning about ethnolinguistic identities to rapidly changing social landscapes, and how inter-individual differences in language competences are themselves a source of intra-population variation in reasoning about linguistic identity.

## Methods

### Study site

In Mexico, Yucatec Mayan is spoken by approximately 759,000 people in the states of Yucatán, Quintana Roo and Campeche, but the country's official national language is Spanish (de León, 2017; INEGI, 2019). Maize swidden agriculture has been the primary mode of subsistence in the Yucatán peninsula since at least the first millennium BC (Aissen et al., 2017 In the villages where this study was carried out (see Padilla-Iglesias et al., in press) fertility is and has traditionally been very high (seven to eight children per woman). The *ejido* system, set up after the Mexican Revolution, establishes that ejido lands cannot be owned, inherited or sold and that their dominion resides within the village collective, which distributes them among married males (Michnowicz, 2015). Consequently, there is little heritable material wealth, leading to life-long monogamy and a very small variance in reproductive success both between males and females and between different males (Kramer, 2005; Kramer & McMillan, 2006).

From the 1980s onwards, rapid socioeconomic changes started to unfold in the region owing to the growth of urban centres, the creation of new roads, a greater availability of schools and increased contacts with Mexican and global cultures (Kramer, 2005). This allowed many individuals, particularly men, to work for wages in nearby Spanish-speaking lowland towns such as Cancun or Tulum (mainly in the tourism sector), while women mostly stayed in the home. However, contrary to what is often regarded as inevitable, this has not triggered an acculturation process or a replacement of subsistence agriculture with market-based jobs (Gurven et al., 2015; Mattison & Sear, 2016). People simply see wage labour as a supplement to agricultural work, in order to increase household productivity in times of need (Padilla-Iglesias et al., in press). Marriages between Mayan and non-Mayan people are very rare and immigration into the region is extremely low (INEGI, 2019).

The schools now available in the villages involved in this study are under the ‘Intercultural Bilingual Education’ legislation (Santibañez, 2016). However, as reported in many other villages in the region (e.g. de León, 2017; Osorio-Vázquez, 2017), in practice, instruction and textbooks are provided only in Spanish. A recent study by Padilla-Iglesias et al. (in press) found that at home too, the directed input children received in Spanish had increased from an average of 21% in 2007 to over 67% in 2014. Nonetheless, Yucatec Mayan was still the dominant language in the villages and preferentially used among adult interlocutors.

### Data collection

The data collected for this study comes from four villages located in the state of Yucatán, about 80 miles to the southwest of Cancun. Structured interviews were undertaken with adults between January and December 2019. The interviews were performed in their homes, and participants could choose whether the questions were asked to them in Spanish or Yucatec Mayan. In the latter case, a local research assistant would ask the questions from a previously verified translation. Both the assistant and the first author were present in all interviews.

#### Relationship between ethnic category membership, language competences and language acquisition

To determine the relationship between the linguistic repertoire of individuals, the way they had acquired them and their perceived ethnic identity, the participants were asked questions across 10 different scenarios, outlined in Table 1.

**Table 1.** Explanation of the different scenarios used for assessing beliefs about the stability, mutual exclusivity and essentialism of linguistic identity

	Scenario ‘On a scale from 0 to 10, how Maya/Mayera would you consider a person if …’
*a*	They were a native Mayan speaker and could only speak Mayan?
*b*	They were a native Maya speaker and bilingual in Spanish?
*c*	They had married a Mayan person and because of that they then learnt how to speak it perfectly?
*d*	They married a Mayan person and because of that they then learnt how to understand Mayan?
*e*	They had Mayan parents but forgot Mayan when they were young?
*f*	They had Mayan parents but could only understand Mayan?
*g*	They had Mayan parents but did not speak or understand Mayan at all?
*h*	They were adopted by Mayan parents at birth and learnt how to understand Mayan?
*i*	They were adopted by Mayan parents at birth and learnt Mayan?
*j*	They were adopted by Mayan parents at birth but only learnt Spanish?

Participants responded on a 0–10 scale, where 0 was ‘not Maya/Mayera at all’ and 10 was ‘very Maya/Mayera’. Half of the participants were assigned to the ‘Maya’ condition and the other half to the ‘Mayera’ one. In a similar manner to Moya and Boyd's (2015) description of the use of the ‘-ista’ suffix when describing ethnic identities in the Peruvian Altiplano, the ‘-era’ suffix in Yucatán is also utilised to denote a Mayan ethnic identity. However, since in Spanish it is normally used when designating a chosen profession or political affiliation, it could lead to less essentialised ratings. Therefore, we wanted to check whether beliefs about ethnolinguistic category membership were consistent and independent of the semantics.

The order in which participants were presented the scenarios was randomised except for the fact that they always received *a* or *b* first in order to facilitate the understanding of the task.

#### Assessment of the acquisition and value of languages

In order to relate the above scenarios with the perceived mode of acquisition of Spanish competences and value of each of the languages, the questionnaire also included the following open-ended questions: ‘Do you consider it more important to learn Mayan, to learn Spanish, or that both are equally important? Why?’ If participants were fluent in Spanish, we asked ‘How do you think you learnt to speak Spanish?’

### Participants

The sample of participants comprised 121 adults (75 females; mean age 35.81, SD 14.87; see Table S1). All were fluent Yucatec Mayan speakers. Their Spanish competences are summarised in Table 1. While the proportion of Spanish speakers did not vary across villages: *χ*^2^ = 10, d.f. = 6, *p* = 0.1, men were much more likely than women to be fluent Spanish speakers (Table 2, *χ*^2^ = 91.6, d.f. = 2, *p* < 0.001).

**Table 2.** Distribution of Spanish level of the *n* = 121 adults who participated in this study. See ESM1 for details on the coding of the level

Spanish level	Females	Males
None (0)	21.4% (*n =* 18)	4.3% (*n =* 2)
Only understand (1)	35.7% (*n =* 30)	30.4% (*n =* 14)
Fluent (2)	42.9% (*n =* 36)	65.2% (*n =* 30)
Total	100% (*n =* 84)	100% (*n =* 46)

ESM, Electronic supplementary material.

### Statistical approach

We fitted Bayesian mixed models with a cumulative link function in order to examine how a Mayan or Mayera identity was related to language acquisition and language competences in Yucatec Mayan and Spanish (see ESM5 for a justification of the use of Bayesian methods).

Since participants responses were given in the form of ratings on a 10-point scale, instead of treating ordered categorical outcomes as continuous predictors, this type of model does not assume that the distance between two values is necessarily the same: it may take more ‘effort’ to move from a Maya rating of 9 to one of 10 than from one of 4 to 5 (McElreath, 2015). Any associated predictor variable, as it increases, moves predictions progressively through the categories in sequence. With a cumulative link function, the cumulative probability of a value (in this case of each ordered rating category from 0 to 10 in the response scale) is the probability of that value or any smaller value. In the present context, the cumulative probability of a rating of 5 is the sum of the probabilities of ratings of 5, 4, 3, 2 and 1. Since ordered categories by convention begin at 1 (a result less than 1 has no probability at all) we transformed the initial 0–10 response scale into a 1–11 scale.

Four different models were fitted with different combinations of predictor variables (see ESM5 for details on model fitting and model comparison). These included a null model (intercept only), and a control model including the age of the interviewee and whether they were assigned to the ‘Mayera’ condition or not (for those who were not, they were asked ‘How Maya’). In addition, we also built a model including whether the interviewee spoke Spanish or not, and a last one with an interaction between Spanish level and ‘Scenario’ (i.e. scenarios *a*–*j* in Table 4). We did not include the interviewees’ sex as predictor variable given its high correlation with their competences in Spanish, which was our predictor variable of interest, but see Figure S5 for the results of a model including an interaction between an interviewees’ sex and ‘Scenario’ rather than their Spanish level.

Random intercepts for ‘village’ (*α*_VILLAGE_) were included in all models to account for the nested structure of the data and associated clustering (McElreath, 2015). Regularising priors were adopted, which are more conservative than the implied flat priors of non-Bayesian procedures, in order to prevent the model from overfitting the data (McElreath, 2015). Having fit alternative parameterisations for all models, we believe that the results presented below are qualitatively robust to changes in priors.

## Results

### Language acquisition and the value of languages

The first issue is to determine: (a) how participants perceived they had themselves acquired competences in each of the languages; and (b) the subjective value they placed on being fluent on each of them.

Since all but one of the participants were native Yucatec Mayan speakers, they all stated that they had learnt to speak Mayan either from their parents at home or in the village.

In line with most participants’ belief that their competences in Spanish had been mainly acquired in school (Table S3), the gendered difference in Spanish competence can be attributed to men's disproportionate access to education: the mean number of years spent in education were 6.14 (SD = 3.74) and 8.33 (SD = 4.01) for females and males respectively.

All of the respondents believed competences in both languages to be equally valuable. The reasons why interviewees ascribed importance to each of the languages are summarised in Table S4.

### Ethnolinguistic reasoning

**Figure 1.**
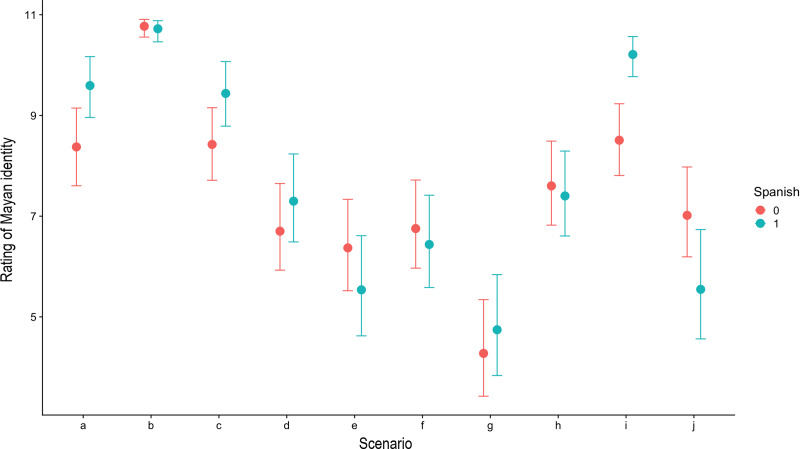
Average response values for each scenario in the full model comprising an interaction between speaking Spanish and ‘scenario’, setting the random intercept for village to 0. Points indicate medians and error bars the 90% HPDIs from the posterior distribution. Blue bars and dots represent respondents who were fluent Spanish speakers and pink bars and dots those who were not.

Our aim here was twofold: first, we wanted to evaluate the extent to which people perceived linguistic differences to denote differences in ethnic identities, and how they reasoned about their inheritance, stability through life and mutual exclusivity with other such identities. That would allow us to determine whether people were using others’ linguistic competences as cues of ethnic category membership and/or relatedness. Second, we wanted to assess whether individual attributes, in particular one's own linguistic repertoire was a source of intra-population variation in reasoning about ethnolinguistic phenomena. To facilitate interpretation of the results reported in the text below and Figure 1, the predictions from each ethnic phenomenon under test are shown in Table 3 together with whether they were met or not, which was determined by assessing the overlap between the 90% HPDIs corresponding to each phenomenon.

**Table 3.** Predictions made by the different ethnic phenomena under test. The first row concerns a general prediction over whether language is somehow associated with people's perception of others’ ethnic category membership. Scenario ‘a’ represents the baseline for comparison. The ‘Prediction’ column indicates the expected relative rating of particular scenarios in the case where people reasoned about ethnolinguistic identities as indicated in the possibilities column. The meaning of the scenarios concerned is explained in the ‘Explanation of prediction column’. A prediction was considered fulfilled when the Highest Posterior Density Interval (HPDI) of the scenarios involved did not overlap, and hence, were significantly different in the expected direction

Ethnic reasoning phenomenon	Possibilities	Prediction	Explanation of prediction	Supported
Overall relationship between ethnic and linguistic identity	Ethnic identity tied to linguistic behaviour	*a* > *g*	Yucatec Mayan speakers should be considered more Mayan/Mayera than those with Mayan parents who do not speak the language	Yes
Heritability	Biologically inherited (genetic)	*c < a* *e, f = a* *f > h* *g > j*	Children with Mayan biological parents should be considered more Mayan/Mayera than those with adoptive Mayan parents	No
	Vertically transmitted (not necessarily biologically)	*i = a* *i, a > c*	Children who learnt Yucatec Mayan after being adopted by Mayan parents should be considered more Mayan/Mayera than those who learnt Yucatec Mayan after marrying a Mayan person	No
	Non-heritable, competency based	*c > d* *a = b = c = i* *d = f = h*	People should regard those who speak Yucatec Mayan as Mayan/Mayera regardless of whether their parents were Mayan or not	Yes*
	Non-heritable, horizontally transmitted	*c = d*	Marrying a Mayan person would make someone Mayan/Mayera regardless of whether they are fluent in Yucatec Mayan	No
Mutual exclusivity	Compatible with competency in Spanish	*a <= b*	Yucatec Mayan–Spanish bilinguals should be regarded as Mayan as Yucatec Mayan monolinguals	Yes
	Not compatible with other identities	*a > b*	Yucatec Mayan–Spanish bilinguals would be regarded as less Mayan/Mayera then Yucatec Mayan monolinguals	No
Stability	Fixed, stable through life	*e = a* *e = g*	Someone with Mayan parents who forgot the language when they were young should be rated as Mayan/Mayera as someone who is a native Yucatec Mayan speaker	No?**
	Flexible, under individual control	*b = c = i* *d = f = h*	Someone who acquires Yucatec Mayan in adulthood should be rated as Mayan/Mayera as someone who acquired the language in childhood	Yes

*b was rated significantly higher than both *a* and *c*. If anything, this goes further against predictions of an essentialisation of ethnolinguistic category membership.

**Since Mayan monolinguals rated *e* > *g*, *a priori* this indicates a perception of some degree of stability throughout the lifetime as even if someone forgot Mayan, they would still be rated as higher in Mayan identity than someone who never learnt the language at all. However, the result may also be due to the fact that many respondents could not believe that someone with Mayan parents would ‘forget’ how to speak Mayan (see Moya & Boyd, 2015 for similar findings).

Ethnic category membership was not perceived as being vertically inherited, genetically or otherwise (Figure 1). Instead, people believed that someone would acquire a Mayan/Mayera identity by becoming fluent in Yucatec Mayan. This was illustrated by the fact that respondents judged that someone who was competent in Mayan, yet had acquired the language in adulthood through marrying a Mayan person, would be as Mayan as someone born into a Mayan family or adopted into it. At the same time, someone who was not fluent in the language, regardless of the identity of their parents, would be regarded as significantly less Mayan than those who were.

Regarding our second aim, the model with the interaction between speaking Spanish and Scenario significantly outperformed all other models (Table 4). This means that the rating given by Spanish and non-Spanish speakers differed across scenarios (Figure 1; see ESM2 for scenario-specific predictions for Spanish and non-Spanish speakers). Nevertheless, unlike in the previously mentioned Peruvian study (Moya & Boyd, 2015), overall participants’ ratings were not influenced by their age (log-cumulative odds = 0.00, 95% HPDI: [−0.01, 0.01]) or by whether they were assigned to the Mayera or Maya condition (log-cumulative odds = 0.25, 95% HPDI: [0.00, 0.50]). Neither did participants differ in their average responses according to their village of residence (estimates for village-specific intercepts were all nearly symmetrical around 0).

**Table 4.** Comparison of ordered-logit models assessing people's perception of others’ ethnic category according to their acquisition of linguistic competences

Model type	WAIC	Weight	SE	dSE
Spanish level × Scenario	4499.1	1	64.61	NA
Spanish level	4533.3	0	62.65	15.76
Control (scenario + age + Mayera)	4536.3	0	62.52	16.49
Intercept only	5063.5	0	43.24	46.07

WAIC, Widely Applicable Information Criterion.

In line with the fact that all of the respondents believed competences in both languages to be equally valuable, being fluent in Spanish was not regarded as reducing someone's perceived Mayan ethnic affiliation. In particular, monolingual Mayan speakers rated as *more* Mayan someone who was bilingual as opposed to monolingual in Mayan (Figure 1).

## Discussion and conclusion

These data show that, although competences in Yucatec Mayan were strongly tied to perceiving others as having a Mayan ethnic identity, ethnolinguistic category membership was *not* essentialised with regard to its stability through life nor perceived as vertically (genetically or otherwise) transmitted nor incompatible with acquiring competences in Spanish.

Children born to Mayan parents were not regarded as more Mayan than those adopted by Mayan parents. Moreover, neither of them would be considered fully Mayan unless they were also competent in Yucatec Mayan. Consequently, we do not find evidence to support the claim that humans have *evolved* to *inevitably* use linguistic boundaries as reliable signals of biological descent, because they are *hard-to-fake,* relatively stable through life or mutually exclusive with one another (e.g. Dunbar, 2003; Richerson et al., 2016; Henrich, 2017; Currie & Mace, 2012). Rather, while there may be an underlying tendency for humans to use language as a marker of ethnic affiliation, it is contingently applied. This flexibility in reasoning about ethnolinguistic category membership makes evolutionary sense; unlike in the Western, industrialised settings where evolutionary psychological research is most commonly carried out (Henrich et al., 2010), cross-culturally and for the greatest part of our species’ history, children tend to spend significant amounts of time with non-related allomaternal carers (carers besides mother) (Kramer & Veile, 2018; Sear & Mace, 2008; Hrdy, 2005). This would entail that a significant portion of children's early cultural (including linguistic) models would not be related to them by recent descent (Lew-Levy et al., 2017, 2018; Migliano et al., 2017; Koster et al., 2019). In such contexts, it would not pay off for humans to have evolved solely to use a shared linguistic repertoire as a proxy of biological relatedness, although it is likely to be part of a battery of proxies (Moya, 2013; Moya & Boyd, 2015).

At the same time, respondents believed that a child adopted into a Mayan family or a person that married a Mayan individual *would* become Mayan *if* they spoke the language. This indeed suggests that individuals may have been using language acquisition as a proxy of the acquisition of other Mayan cultural norm clusters, and thus of ethnic affiliation. Importantly, however, such ethnic category membership was not regarded as vertically inherited or incompatible with acquiring competences in Spanish, and thus presumably majority cultural norms.

However, there is also extensive evidence that cultural differences can guide genetic evolution over relatively short periods (Henrich, 2017; Tishkoff et al., 2007). Thus, it could be that selection has been differentially operating on how people reason about ethnolinguistic boundaries across populations according to the environment experienced by previous generations in those same settings. The newness of bilingualism in Yucatán allows us to assess whether individuals respond to changes in ethnolinguistic landscapes over periods that are significantly shorter for evolution to take place, and thus rule out that possibility (Kramer, 2005; Gaskins, 2003). Our results add to previous work suggesting that the observed inter-cultural and inter-individual differences in reasoning about linguistic identity, and/or using language as means to guide particular social behaviours, are the result of evolved developmental mechanisms that are sensitive to cultural influence (Kline et al., 2018). In other words, that our development is plastic enough that humans can socially learn how to use social information around them, and that this leads to psychological and behavioural variation both within and across populations (Mesoudi, et al., 2016). Accordingly, when the different languages available to individuals serve different social functions, they may reason about and use each of them differently. For example, the Wichí of Argentina believed a Wichí and a Chorote identity (both indigenous Native American groups) to be compatible and dependent on acquiring the language associated with each of the groups early in life (Erut, 2017). However, they did regard holding a Wichí and a Criollo (‘white’, non-indigenous) identity (and the languages associated with them) to be mutually exclusive, indicating that the way in which people reason about the compatibility of multiple ethnic identities is related to the social relationship of the involved groups.

In the Yucatec Mayan context, given that the possibilities for monetising crops are limited, as is the land available for cultivation (Schacht et al., 2018), there is a plateau in how much each family member can contribute to household productivity were households rely exclusively on farm work. At the same time, since entry into the marketplace requires particular linguistic skills (i.e. Spanish) but also substantial time investment away from the village that competes directly with the ability to employ traditional means of obtaining food, it is common for *some* family members to participate in wage labour to supplement household productivity. Consequently, the languages serve different, yet adaptive social functions – Mayan granting access to the communally owned land and the associated networks of exchange of resources, childcare and labour, and Spanish as a useful tool to access out-group networks and the economic opportunities they offer. This means that potentially everyone can benefit from a non-mutually exclusive linguistic identity.

Humans may have evolved to pay special attention to language when reasoning about others’ cultural identities. In the present setting, this was evidenced by the fact that, even if ethnolinguistic categories were regarded as fluid, not vertically inherited and not essentialised with respect to identity stability or mutual exclusivity, language competences *were* used as proxy of ethnic category membership when no other information was available. However, rather than a fixed default (e.g. Cohen, 2012; Dunbar, 2003), our development is flexible enough to allow differences in the cultural norm clustering that occur around linguistic boundaries, to lead to culture-specific and language-specific ways in which people reason about the relationship between language and ethnic identity (e.g. Moya & Scelza, 2015; Moya & Boyd, 2015). Support for this view comes from communities situated in the Quechua–Aymara linguistic boundary in Peruvian Altiplano, where children below 9 years of age saw linguistic identities as more fixed, but adults did not (Moya et al., 2015). Therefore, even if new-borns may start off with a prior expectation that language is predictive of others’ ethnic identities, rather than it representing a ‘core cognitive system’ for ‘dividing the social world into *us* vs. *them*’ (cf. Kinzler and Spelke, 2007), developmental plasticity allows them to update such beliefs to adapt to the environments where they grow up. In fact, far from a binary division, our results show that ethnic category membership was conceived in terms of *degrees*, which could be strategically altered by social context and personal attributes, illustrated by participants’ use of the full range of the response scale. Future research is still needed to determine how strong the expectation that linguistic category membership carried useful social information was.

The flexible nature of human cognitive systems was also illustrated by intra-population variation in the way people reasoned about ethnolinguistic identities according to their *own* socially acquired characteristics (in this case their linguistic repertoires) (Cohen & Haun, 2013). In this study, not only were bilinguals generally not regarded as less Mayan, but particularly Mayan monolinguals gave them a higher rating in their Mayan ethnic identity than those individuals who only spoke Mayan. One possibility is that since a lot of bilinguals serve as bridges between urban towns and the local communities, they are seen as ‘active participants’ in the village, and as key for the structuring the Mayan mixed economy. For example, 43.37% (*n* = 36) of the women in the sample mentioned that they required the assistance of bilinguals to accompany them whenever they needed to go to the doctor in order to help them communicate. This, in turn, may make them be seen as deserving of such a high rating in their Mayan identity. This is also supported by the fact that all respondents valued bilingual acquisition (as opposed to monolingualism in either language).

Mayan monolinguals also appeared to essentialise ethnolinguistic identity with respect to its stability through life as they gave higher ratings to those who had forgotten how to speak Mayan compared with those who had never learnt it at all (Figure 1). Similar phenomena have also been observed among Canadian children, where sequential bilinguals (children who had acquired a second language after 3) showed reduced essentialist beliefs with regards to language category membership than monolinguals and simultaneous bilinguals, presumably because they were more likely to understand that languages are learnt through experience (Byers-Heinlein & Garcia, 2015).

Last, understanding the power of socioeconomic changes to redefine the relationship between ethnic and linguistic identity is also particularly important in order to assess the prospect of linguistic diversity persisting in the future (Pietikäinen, 2018). The kind of reasoning about others that people make based on their linguistic profiles can have severe consequences for the social behaviours they display towards them. Research has shown that adults who hold stronger essentialist beliefs are more likely to endorse stereotypes (Bastian & Haslam, 2006) and prejudiced attitudes (Haslam, 2002) or be less willing to compromise with outgroups (Halperin et al., 2011).

**Figure 2.**
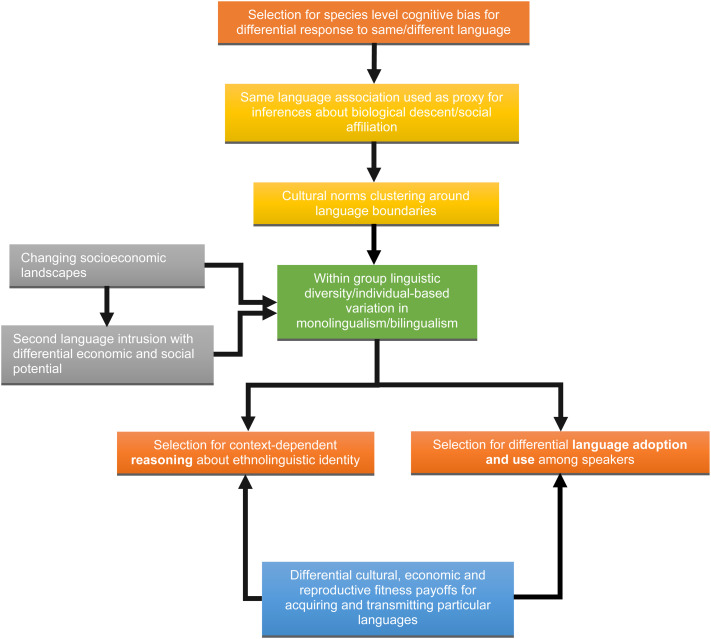
Schematic representation of the processes linking reasoning about linguistic identity, language-based social behaviours and the payoffs to bilingual acquisition following socioeconomic changes. The ochre boxes represent species-specific tendencies to use elements of language as markers of ethnicity or other forms of social affiliaiton. The grey boxes indicate the changing socioeconomic circumstances which occur with the introduction of a second language. The outcome is within-group diversity in language use (green box). This more varied linguisitc context will lead to selection for both diffential language use among speakers, and reasoning about ethnolinguistic identity (orange boxes). The differential outcomes of both the adoption of second languages and reasoning about their use will be the payoffs (blue box).

In conclusion, we found evidence that speaking Mayan was not regarded as a proxy of biological relatedness but was regarded as a badge of membership to the cultural group – that is, as marker of a Mayan ethnic identity. This was not incompatible with acquiring competences in Spanish, which was instead simply seen as a functional tool to access the new opportunities offered by increased connectedness with urban centres, including wage labour, healthcare and education. By highlighting the plasticity of the psychological underpinnings of our way of reasoning about linguistic category membership, our study suggests that when different languages serve different yet adaptive social functions and encompass different cultural norm clusters, individuals may reason about them differently. In turn, this means that we should expect the way people to reason about what linguistic affiliation means to be plastic and context specific even if built on an evolved universal human psychology (Figure 2). Such plasticity becomes especially important in the context of rapidly changing conditions, particularly when these involve exposure to new languages, but it would be important to also study contexts where bilingualism may have a longer and more stable history. Furthermore, we have focused here on the context-sensitive and flexible nature of reasoning about language use, and such more stable contexts may provide a better insight into how this may articulate with the evolved capacity to see language as an ethnic marker. How people reason about linguistic identity in different circumstances will condition the social behaviours that they display towards others who hold the same/different ones and therefore the payoffs to acquiring and transmitting particular languages.

## Data Availability

The data used in this study are available as Supplementary Files.

## Supplementary material

For supplementary material accompanying this paper visit http://dx.doi.org/10.1017/ehs.2020.39. click here to view supplementary material
