# Supplementary material for: Language as a marker of ethnic identity among the Yucatec Maya
Source: Evol Hum Sci. 2020 Jun 29;2:e38. doi: 10.1017/ehs.2020.39 (PMC10427450; doi:10.1017/ehs.2020.39)
Supplement: Supplementary file 1 [file ehssup.zip › S2513843X20000390sup001.docx]

**Appendix. Questionnnaire used for the interviews in Spanish (top) and Yucatec Mayan (bottom)**

En una escala de 0 a 10, ¿Hasta que punto es una persona Maya si:

- 1. Su idioma nativo es Maya y solo puede comunicarse en ese idioma
  2. Su idioma nativo es Maya y es bilingüe en español
  3. Se casaron con un Maya y luego de lo cual aprendieron Maya
  4. Se casaron con un Maya, luego de lo cual logran comprender el Maya
  5. Tienen padres Mayas, pero olvidaron el idioma cuando eran jóvenes
  6. Tienen padres Mayas, pero solo logran comprender el idioma
  7. Tienen padres Mayas, pero no hablan el idioma en lo absoluto
  8. Fueron adoptados por padres Mayas al nacer y aprendieron a comprender el Maya
  9. Fueron adoptados por padres Mayas al nacer y aprendieron Maya
  10. Fueron adoptados por padres Mayas al nacer, pero solo aprendieron español

Ich tak 0 tak 10, ¿Buká u mayajil un tu’ul mak wa:

- 1. U yax t’an jach Maya tsokole chen yetel ku beytal u tsikbal
  2. U yax t’an jach Maya tsokole ku t’anik tak español
  3. Tsok u bel yetel un tuul Maya, tsokole ka tu kana maya
  4. Tsok u bel yetel un tuul Maya, tsokole ka tu najtá le Maya
  5. Yan u papa jach maya, pero ka nuklajobe ka tu tuubsó
  6. Yan u papa jach maya, pero chen ku najtikoob
  7. Yan u papa jach maya, pero jach mix u p’it ku najtikoob
  8. Chen mataboob tumen maya winik tu chichniloob ku najtikoob Maya
  9. Chen mataboob tumen maya winik tu chichniloob yetel tu kanaj Maya
  10. Chen mataboob tumen maya winik tu chichniloob, pero che español tu kanaj
