# Supplementary material for: Language as a marker of ethnic identity among the Yucatec Maya
Source: Evol Hum Sci. 2020 Jun 29;2:e38. doi: 10.1017/ehs.2020.39 (PMC10427450; doi:10.1017/ehs.2020.39)
Supplement: Supplementary file 1 [file ehssup.zip › S2513843X20000390sup003.pdf]

<u>**How reasoning about language constructs a flexible ethno-linguistic categorization system: A case study among the Yucatec Maya.**</u>

By Cecilia Padilla-Iglesias, Robert A. Foley and Laura A. Shneidman

<u>**Electronic Supplementary Material**</u>

ESM1. Coding of language competences

In the descriptive statistics, a Spanish level of 0 indicates not being able to understand or speak the language at all; 1 indicates an ability to understand it and speak a little bit; 2 indicates ability to speak the language fluently.

For all participants of household, Spanish level was assigned after asking them a) whether they spoke Spanish, b) how well, c) whether they understood the doctor when he/she spoke in Spanish, d) whether they understood telenovelas that were in Spanish, e) whether they required help in order to translate what the doctor was saying (as health clinics tend to be located in Spanish-speaking urban centres).

For fitting the Bayesian multilevel models with cumulative link function, we binary coded Spanish level, with 0-1 in the original scale being coded as 0 and 2 being coded as 1 (hence only individuals that are sufficiently fluent in Spanish are coded as Spanish speakers).

ESM2. Descriptive statistics

Table S1: Overview of the socioeconomic profiles of the n=121 participants of the study

	**Percentage females**	**Percentage males**
Are married	92.9% (n=78)	67.4% (n=31)
Were born in current town of residence	50% (n=42)	82.6% (n=38)
Have ever lived in a city	29.8% (n=25)	23.9% (n=11)
Are worried about not having enough money to feed their family that month	57.1% (n=48)	56.5% (n=26)
Engage in wage labour outside their town of residence	13.1% (n=11)	60.9% (n=28)

ESM2. Coefficients of the full (interaction) model and condition-specific plots

Table S2: Coefficients from the full Bayesian multilevel model (comprising an interaction between the interviewee's competences in Spanish and "Condition") with cumulative link function predicting the rating in Maya or Mayera identity across conditions.

Random Effect	Term	Estimate	95% HPDI ll	95% HPDI ul
	b_Intercept.1.	-4.07	-4.86	-3.29
	b_Intercept.2.	-3.95	-4.75	-3.18
	b_Intercept.3.	-3.62	-4.39	-2.83
	b_Intercept.4.	-3.32	-4.11	-2.56
	b_Intercept.5.	-3	-3.79	-2.25
	b_Intercept.6.	-1.9	-2.66	-1.14
	b_Intercept.7.	-1.43	-2.2	-0.69
	b_Intercept.8.	-0.84	-1.58	-0.08
	b_Intercept.9.	0	-0.77	0.74
	b_Intercept.10.	0.53	-0.2	1.31
	b_Conditionb	2.08	1.38	2.82
	b_Conditionc	-0.3	-0.88	0.34
	b_Conditiond	-1.65	-2.26	-1.07
	b_Conditione	-1.8	-2.43	-1.18
	b_Conditionf	-1.71	-2.3	-1.11
	b_Conditiong	-3.17	-3.81	-2.49
	b_Conditionh	-1.03	-1.62	-0.43
	b_Conditioni	-0.24	-0.83	0.36
	b_Conditionj	-1.59	-2.21	-0.98
	b_Age_c	0	-0.01	0.01
	b_Mayero	0.25	0	0.5
	b_Spanish	0.57	-0.09	1.18
	b_Conditionb.Spanish	-1.81	-2.76	-0.89
	b_Conditionc.Spanish	-0.3	-1.11	0.55
	b_Conditiond.Spanish	-0.75	-1.55	0.04
	b_Conditione.Spanish	-1.42	-2.29	-0.62
	b_Conditionf.Spanish	-1.1	-1.88	-0.27
	b_Conditiong.Spanish	-0.94	-1.78	-0.07
	b_Conditionh.Spanish	-1.05	-1.84	-0.23
	b_Conditioni.Spanish	0.63	-0.17	1.48
	b_Conditionj.Spanish	-2.27	-3.12	-1.42
(Intercept)	Town_id.1	-0.15	-0.82	0.37
(Intercept)	Town_id.2	0.14	-0.44	0.8
(Intercept)	Town_id.3	-0.32	-0.99	0.23
(Intercept)	Town_id.4	0.39	-0.2	1.15

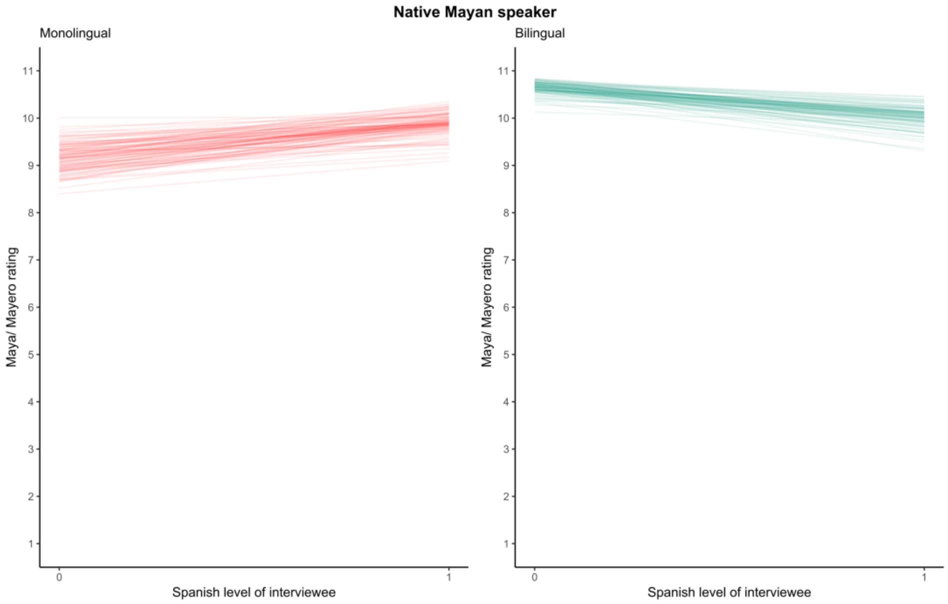

Fig. S1: Predictions from 10,000 samples from the posterior distribution of the full ordered categorical model with an interaction between Spanish level and condition. Left-right the plots show how the distribution of predicted responses for conditions “a” and “b” in the main text (respectively) vary depending on the Spanish level of the interviewee.

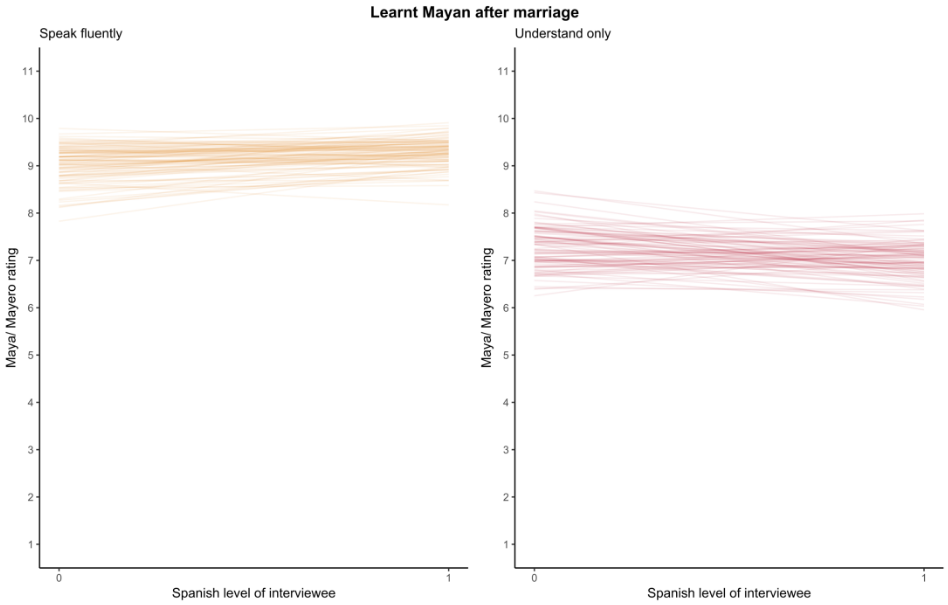

Fig. S2: Predictions from 10,000 samples from the posterior distribution of the full ordered categorical model with an interaction between Spanish level and condition. Left-right the plots show how the distribution of predicted responses for conditions “c” and “d” in the main text (respectively) vary depending on the Spanish level of the interviewee.

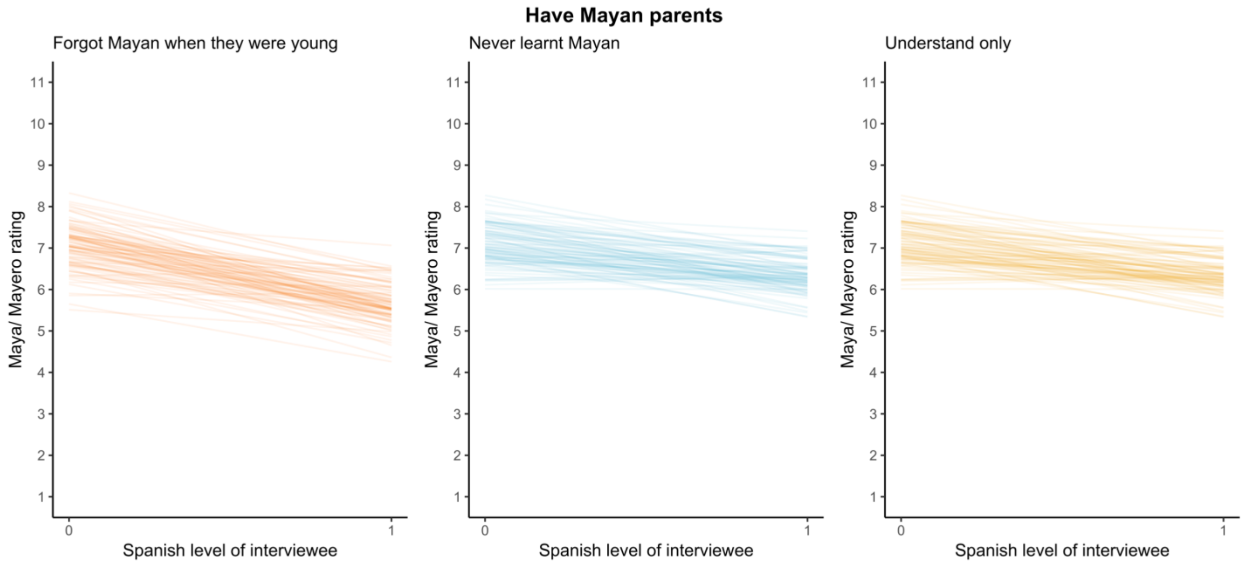

Fig. S3: Predictions from 10,000 samples from the posterior distribution of the full ordered categorical model with an interaction between Spanish level and condition. Left-right the plots show how the distribution of predicted responses for conditions "e", "f" and "g" in the main text (respectively) vary depending on the Spanish level of the interviewee.

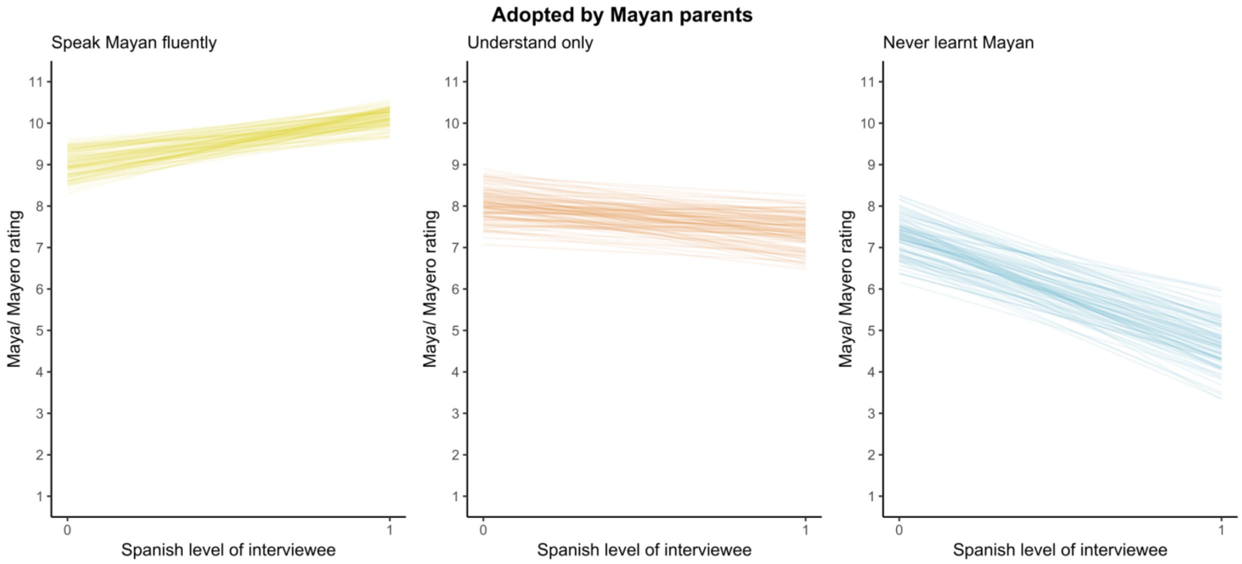

Fig. S4: Predictions from 10,000 samples from the posterior distribution of the full ordered categorical model with an interaction between Spanish level and condition. Left-right the plots show how the distribution of predicted responses for conditions "i", "h" and "j" in the main text (respectively) vary depending on the Spanish level of the interviewee.

ESM3. Predictions from alternative model including an interaction between participants' sex and "Scenario" as predictor variables.

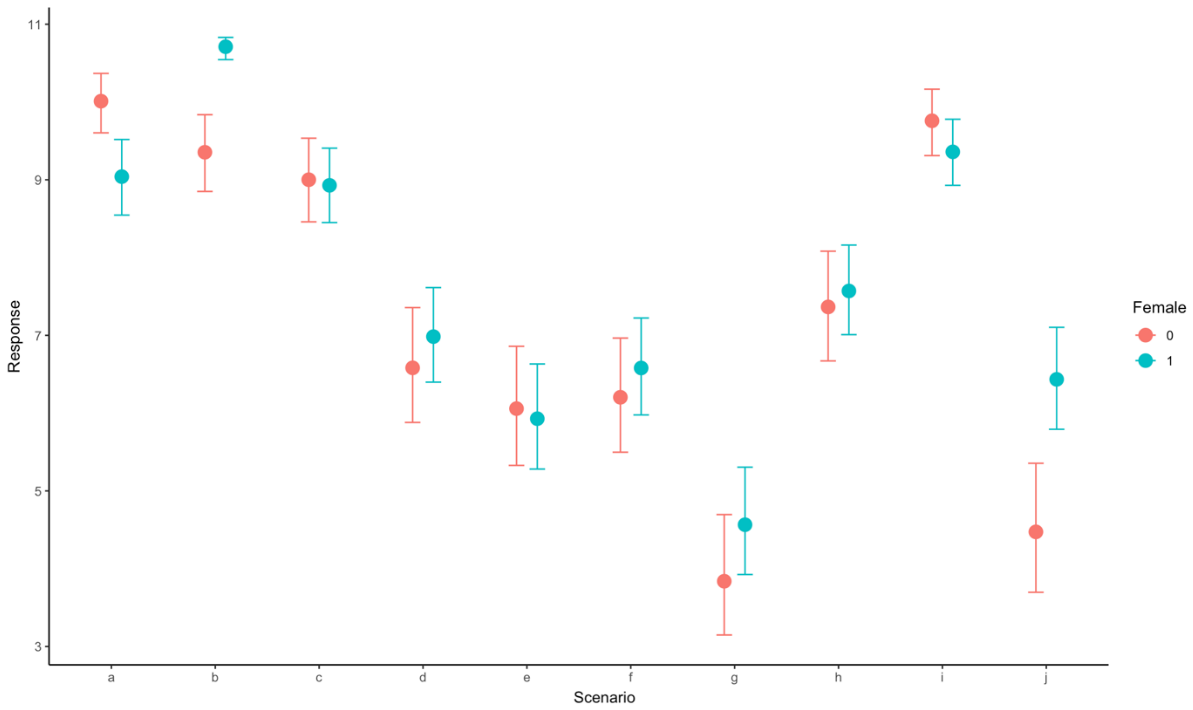

Fig. S5: Average response values for each condition in the model comprising an interaction between "sex" and "condition" in addition to the control variables (participants' ages and whether they were assigned to the Maya or Mayera condition) setting the random intercept for village to 0. Point indicates median and error bars the 90% HPDIs from the posterior distribution. Blue bars and dots represent female respondents and pink bars and dots represent males.

ESM4. Qualitative descriptions of perceived ways in which participants had acquired competences in Spanish

Table S3: Self-reported beliefs about Spanish acquisition by the 94 adults that were fluent Spanish speakers. The fact that the total is greater than 66 is because some women gave more than one answer to the question.

Question	Answer	Count
**How did you learn Spanish?**	School	65
	Worked in city (Valladolid/Cancun)	9
	Were born in a city (Valladolid/Cancun)	3
	From their children	4
	From visiting cities	3
	Parents talked to them in Spanish	7
	From listening to others	3
	**TOTAL**	94

ESM5. Advantages of Bayesian inference and details on model fitting

In a Bayesian framework, a model conditions its data on prior probability distributions and uses Monte Carlo sampling methods to generate posterior distributions for its parameters. The priors are the initial probabilities for each possible value of each parameter. This permits comparisons between posterior distributions across age groups, villages or linguistic categories without requiring specific post-hoc tests and obviates the need to adjust for multiple comparisons (Gelman et al., 2012). Bayesian inference also allows a better interpretation of differences between parameter estimates relative to a specific value by obtaining the entire posterior distribution for each predictor and showing the highest posterior density intervals (HPDIs), that reveal the narrowest interval containing the specified probability mass.

Our models took the form:

$R_i \sim$ Ordered-logit(φ_i, κ)	[Probability of the data]
$\varphi_i = 0 + \alpha_{VILLAGE} - \beta S_i$	[Linear model]
$\kappa_k \sim$Normal(0, 1.5)	[Common prior for each intercept]
$\alpha_{VILLAGE} \sim$Normal(0, 10)	[Prior for unique intercept per village]
$\beta \sim$Normal(0, 2)	[Prior for β]

The log-cumulative-odds of each response *k* was defined as a sum of its intercept α_k and a typical linear model. The linear model φ is subtracted from each intercept because if the log-cumulative-odds of every outcome value *k* is decreased below the maximum, this necessarily shifts the probability mass upwards towards higher outcome values.

Before the analysis, we checked for multicollinearity among predictors using the generalized variance inflation factor (GVIF). All GVIF values fell below the lowest commonly recommended threshold of 2, indicating that none of the models should suffer from multicollinearity (Zuur et al. 2010).

Parameter estimation was achieved with RStan (Stan Development Team, 2016), running three Hamiltonian Monte Carlo chains in parallel, and obtaining 10,000 samples from each, 2,000 of which were used as warm-up. Convergence was verified by a high effective number of samples and R^ estimates of 1.00 (McElreath, 2015). We also visually inspected trace plots of the chains to ensure that they converged to the same target distributions and compared the posterior predictions to the raw data to ensure that the models corresponded to descriptive summaries of the samples. For model comparisons, we used Widely Applicable Information Criteria (WAIC) which provides an approximation of the out-of-sample deviance that converges to the leave-one-out cross-validation

approximation in a large sample (Gelman et al., 2013). Analyses were performed in R 3.5.2 using the *brms* package (R Core Team, 2018; Bürkner, 2017).

Supplementary References

Bürkner, P.-C. (2017). brms: An R package for Bayesian multilevel models using Stan. *Journal of Statistical Software*, *80*(1), 1–28.

Gelman, A., Carlin, J. B., Stern, H. S., Dunson, D. B., Vehtari, A., & Rubin, D. B. (2013). *Bayesian data analysis*. Chapman and Hall/CRC.

Gelman, A., Hill, J., & Yajima, M. (2012). Why we (usually) don't have to worry about multiple comparisons. *Journal of Research on Educational Effectiveness*, *5*(2), 189–211.

McElreath, R. (2015). *Statistical rethinking: Texts in statistical science*. Boca Raton, FL: CRC Press.

R. Core Team. (2018). *R: A language and environment for statistical computing.*

Stan Development Team. (2016). Stan modeling language users guide and reference manual. *Technical Report*.

Zuur, A. F., Ieno, E. N., & Elphick, C. S. (2010). A protocol for data exploration to avoid common statistical problems. *Methods in Ecology and Evolution*, *1*(1), 3–14.
